# Images in clinical medicine: Popeye's sign

**DOI:** 10.3402/jchimp.v3i2.20688

**Published:** 2013-07-05

**Authors:** Shobhit Gupta, Andy Crocker, Jorge Scheirer, Richard Alweis

**Affiliations:** 1 Department of Medicine, Reading Health System, West Reading, PA, USA; 2 Department of Medicine, Jefferson Medical College, Philadelphia, PA, USA

**Fig. 1:**
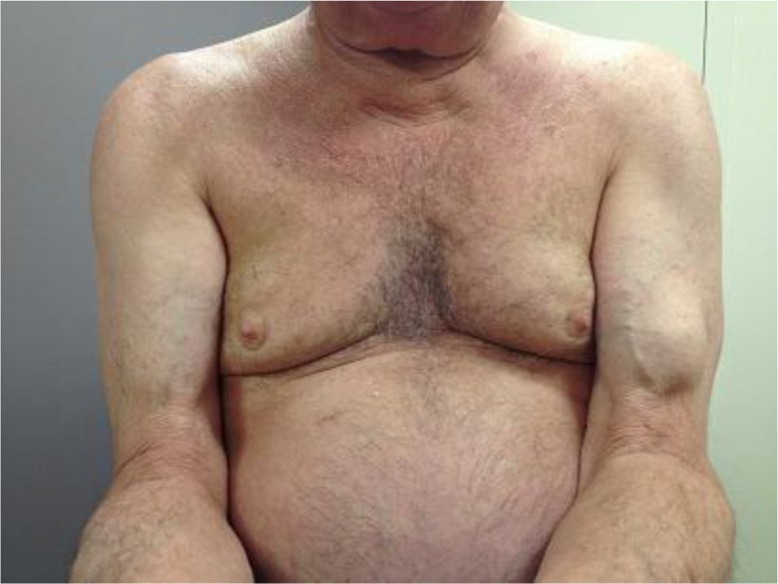
Left arm mass also known as ‘Popeye's’ sign.

A 72-year-old male with a history of bilateral rotator cuff tears presented to his primary care physician (PCP) with complaints of chronic shoulder pain and fatigue. Upon physical examination, a palpable mass was noted between his left shoulder and elbow, although he could not recall when the mass first appeared. Left arm cogwheel rigidity was also noted on physical examination. He denied any tenderness to the left shoulder, elbow, or previous mass palpation. The location of the mass is consistent with Popeye's sign, a hallmark of biceps tendon rupture (Fig. 1). He was referred to an orthopedic surgeon for evaluation but opted instead to strengthen his shoulder through physical therapy.

The biceps brachii muscle primarily functions as a forearm supinator (1). The muscle is formed by a long and short head that share a distal tendon attachment site, but have distinct proximal tendon attachment sites on the scapula (2). Rupture of the biceps tendon can occur at either the proximal or distal sites, although distal rupture is rare (1). The mechanism of injury can range from degenerative changes in the elderly to athletic injuries related to weight lifting or throwing in younger patients. Trauma at any age can result in a biceps tendon rupture, and these injuries are often associated with a ‘popping’ sound. Clinical manifestations of ruptures often include a sudden onset of sharp shoulder pain, ecchymosis, and swelling (3). In patients with chronic shoulder pain, however, an acute rupture may improve pain quality (3). As noted in Fig. 1, acute tendon rupture is classically associated with the ‘Popeye’ deformity, a visible or palpable mass present near the elbow or in the mid-upper arm (3). Diagnosis of a biceps tendon rupture is primarily made by history, inspection, and palpation. Imaging modalities such as MRI or ultrasound may be helpful to delineate a complete versus partial rupture, but are otherwise unnecessary to make a diagnosis (1). There are no consensus guidelines on indication for surgical repair, although distal ruptures seem to have greater clinical benefit from repair as these patients have significant defects on their physical examination (1). Chronic tears become increasingly more challenging to treat due to possible tendon contraction and poor tissue quality but tendon grafts have been successfully used for these cases (1). Active patients who depend on arm strength, such as athletes, may be appropriate candidates for surgical referral.
